# Unveiling the Structure of Cognitive Vulnerability for Depression: Specificity and Overlap

**DOI:** 10.1371/journal.pone.0168612

**Published:** 2016-12-16

**Authors:** Igor Marchetti, Tom Loeys, Lauren B. Alloy, Ernst H. W. Koster

**Affiliations:** 1 Ghent University, Ghent, Belgium; 2 Temple University, Philadelphia, United States of America; Istituto Superiore Di Sanita, ITALY

## Abstract

There is extensive literature establishing the influence of rumination, hopelessness, and dysfunctional attitudes on depressive symptoms. However, it is unclear whether these vulnerability factors are distinctly related to depressive symptoms or show substantial overlap. In two large samples of undergraduates (Study #1, n = 304; Study #2, n = 491) and two samples of clinically depressed individuals (Study #3, n = 141; Study #4, n = 109, from published studies), questionnaire data were used to examine the relationship between cognitive vulnerability factors and depressive symptoms, along with additional measures of anxiety and stress symptoms. To decompose model fit into its specific and common partitions, we relied on commonality analysis (CA). CA showed that there is substantial overlap in cognitive risk factors for depression. Moreover, we found strong evidence that hopelessness provides a unique statistical contribution to depression. This pattern of findings was stable in healthy as well as clinical samples. Symptom-levels analysis revealed that a specific subset of depressive symptoms are associated with hopelessness. In closing, we showed that CA provides a powerful tool to map unique and overlapping variance between multiple risk factors. Moreover, hopelessness emerged to be an important focus of clinical attention.

## Introduction

Depression is increasingly being considered a global health priority, with over 298 million cases estimated world-wide in 2010 [1], thus qualifying this disorder as the second global leading cause of years lived with disability [2]. Given this worrisome scenario, it is of paramount importance to shed light on the factors that enhance and maintain the risk for developing depression.

Basic and clinical research indicates that cognitive factors are important etiological and maintaining factors for depression [3]. The findings suggest that negatively valenced, dysfunctional, rigid, and sometimes unrealistic thinking has deleterious effects on mood, motivation, and psychophysiology, eventually resulting in clinical depression [4]. Within the landscape of cognitive models of depression, three major theories have generated extensive and robust evidence supporting their theoretical claims about the origin and persistence of depression.

First, Aaron T. Beck’s model [5–7], the first and most influential cognitive theory of depression, posits the existence of latent negative schemata leading to thinking distortions and multiple cognitive biases (e.g., attention, interpretation, and memory), which eventually enhance the occurrence of depressive symptoms. The key feature of Beck’s model is the existence of negative schemata, defined as internal beliefs or representations of stimuli, ideas, or experiences [4]. As depressogenic cognitive structures, negative schemata are supposed to spur the proliferation of *dysfunctional attitudes*, that is pernicious beliefs such as “*If I fail something*, *it means I’m a total failure*”. Dysfunctional attitudes are typically measured by the *Dysfunctional Attitudes Scale* (DAS) [8]. Beck’s model explicitly proposes, and research has confirmed, that dysfunctional attitudes represent a powerful cognitive vulnerability to depression [3].

Second, the Hopelessness Theory [9] posits that when individuals regularly make global, stable, and internal attributions for negative events, this leads to the development of hopelessness. Hopelessness is defined as the expectations that undesirable outcomes will occur and that desirable outcomes will not occur along with the perceived inability to change this dismal scenario (i.e., *My future seems dark to me*). According to the theory, hopelessness, rather than being a symptom of depression, is a powerful cognitive vulnerability for depressive symptoms, and it is usually measured by means of the *Beck Hopelessness Scale* (BHS) [10]. Hopelessness has proven to be a strong predictor of depression [11].

Finally, the Response Styles Theory [12] highlights that the way individuals react to distress is of critical importance with respect to outcomes of mental well-being. Rumination involves focusing in a passive and repetitive way on the source and the consequence of distress, which invokes self-critical and negative thinking and reduced concrete, problem-oriented thinking. There is ample evidence supporting the role of ruminative thinking in the onset and maintenance of depressive complaints [13]. Importantly, since the development of a key self-report measure (*Ruminative Response Scale*, RRS) [12], more advanced psychometric analysis [14] has identified a specific maladaptive subtype of rumination, defined as *brooding*, that is the trait tendency of individuals to passively focus on symptoms of distress and the meaning of those symptoms (i.e., *“What am I doing to deserve this*?*”*).

However, despite the fact that dysfunctional attitudes, hopelessness, and rumination/brooding predict concurrent and prospective depressive symptoms and major depressive episodes [11, 13, 15–19], the exact nature of how these three major cognitive risk factors are related to one another and depressive symptoms is still unclear. Such lack of clarity arises from both absence of any theory-based taxonomy (for an exception, see [20]) and empirical evidence suggesting mutually-exclusive models [21–23]. For instance, Lam and colleagues [21] found that rumination indirectly predicts depression and hopelessness via mediation of dysfunctional attitudes, while other studies suggest that negative expectations about the future mediate the relationship between rumination/brooding, cognitive vulnerability, and depression symptoms [22, 23].

Therefore, there are multiple ways in which the considered risk factors may interact. Given the moderate-to-strong degree of correlation among the vulnerability factors [24], dysfunctional attitudes, hopelessness, and rumination could be completely redundant with one another in accounting for depressive symptoms. Were this the case, no substantial unique effects could be detected. Alternatively, despite their overlap, specific unique effects of the considered risk factors could still be present and play a major role in explaining depression. To our knowledge, a thorough investigation of the underlying structure of cognitive vulnerability has never been conducted, although this could substantially improve our understanding of psychological vulnerability.

To address this issue, we examined the degree of *specificity* (i.e., unique variance) and *overlap* (i.e., common variance partitions) of dysfunctional attitudes, hopelessness, and ruminative brooding (or rumination) in accounting for depressive symptoms, using commonality analysis [25–27]. In detail, we first report two studies (i.e., Study #1 and Study #2), consisting of healthy and subclinical undergraduate individuals along different depression-related outcome measures (i.e., Study #2a and #2b). In order to test the extension to clinical samples of our findings, we report two further studies (i.e., Study #3 and Study #4a,b), derived from published research (i.e., secondary analysis), with clinical samples [21, 28].

## Methods

### Participants

In Study #1, we recruited 304 undergraduate students (mean age: 20.75 ± 3.22, range: 18–44, 85.5% female; from the original 316 participants, 3.8% were excluded due to missing data; convenience sample) and in Study #2, 491 undergraduate students (mean age: 20.86 ± 3.22, range: 18–46, 85.2% female; from the original 495 participants, 0.8% were excluded due to missing data; convenience sample) from Ghent University. Study #3 [28] listed 141 participants (median age: 41, range: 27–53, 90.1% female) reporting the following diagnoses: 89 = major depressive disorder (MDD); 13 = MDD with comorbid borderline personality disorder, 39 = no psychiatric diagnosis. Study #4 [21] recruited 109 MDD outpatients (mean age: 44.4 ± 12.8, 65.4% female). All the participants actively recruited for this study provided written informed consent. This study and the related consent procedure were approved by the Ethics Committee of the Faculty of Psychology and Education Sciences of Ghent University.

### Materials

Throughout the reported studies, individual levels of depressive symptoms (and in Study #2b, also anxious and stress-related symptoms), rumination/brooding, hopelessness, and dysfunctional attitudes were measured with similar instruments.

To measure *depressive symptoms*, the following instruments were used: Beck Depression Inventory 2^nd^ Edition (BDI-II) [29] in Study #1 (α = .92, mean = 7.72 ± 7.72, range: 0–45) and Study #2a (α = .90, mean = 8.28 ± 7.48, range: 0–46); Depression Anxiety Stress Scales 21 items (DASS-21) [30] in Study #2b (depression subscale α = .86, mean = 2.93 ± 3.61, range: 0–20); Beck Depression Inventory (BDI) [31] in Study #3 and #4a; Hamilton Rating Scale for Depression (HAM-D) [32] in Study #4b. In Study #2b, anxious and stress-related symptoms were measured by means of the related DASS-21 subscales (α = .78, mean = 3.09 ± 3.40, range: 0–20; and α = .87, mean = 5.39 ± 4.41, range: 0–21).

*Rumination* was measured as follows: in Study #1 and #2, Ruminative Response Scale (RRS) [14], more specifically, we operationalized rumination as its most maladaptive component, namely brooding (α = .78, mean = 10.24 ± 3.31, range: 5–20; and α = .76, mean = 10.57 ± 3.23; range: 5–20); in Study #3 and #4, Response Style Questionnaire (RSQ) [12]. However, Lam and colleagues [21] ran a Principal Component Analysis on the RSQ and reported three significant factors. Among these three factors, we included the component (i.e., “symptom-based rumination”) accounting for most variance (i.e., 35.2%) and showing the highest internal consistency (i.e., α = .79) and the highest correlation with depressive symptoms (r = .37, *p* < .001¸ n = 109).

In all the studies, *hopelessness* was measured with the Beck Hopelessness Scale (BHS) [10]; in Study #1 and #2, α = .80, mean = 4.68 ± 3.48; range: 0–19; and α = .82, mean = 4.73 ± 3.62, range: 0–19, respectively) and *dysfunctional attitudes* with the Dysfunctional Attitudes Scale (DAS) [8]; in Study #1 and #2, α = .94, mean = 130.95 ± 28.79, range: 53–242; and α = .92, mean = 129.89 ± 27.52, range: 59–221, respectively).

### Statistical analysis

Throughout the four studies reported, we ran a series of multiple regressions in which individual levels of depressive symptoms were regressed on cognitive risk factors for depression along with additional covariates, if appropriate. We reported Pearson’s zero-order correlations (*r*) among the set of variables and the (un)standardized regression coefficients (*B* and *β*) along with their statistical significance and the amount of variance accounted for by the model (i.e., squared multiple correlation or R^2^). Statistical assumptions, such as linearity, and presence of outliers or out-of-scale scores were checked.

Then, we the investigated the structure of association between the three cognitive risk factors and depressive symptoms by means of commonality analysis. Commonality analysis (CA) is a variance partitioning procedure that allows decomposing model fit (R^2^) into non-overlapping uniquely and commonly explained partitions [25, 26, 33, 34]. By relying on the algebraic product expansion of all the predictors, CA yields partitions that always sum to R^2^ and can be conveniently viewed as effect sizes (e.g., < 1% negligible, > 1% small, > 9% moderate, and > 25% large; [35]).

**Fig 1:**
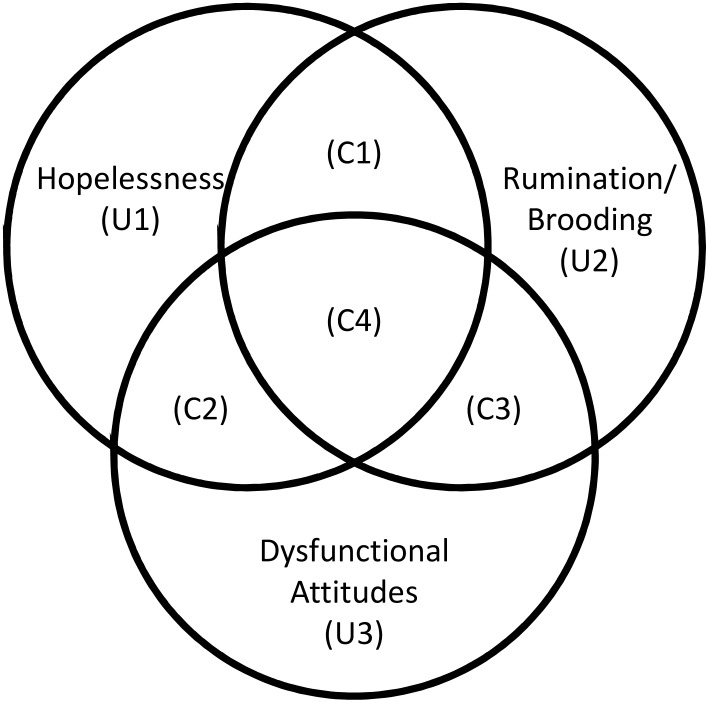
Commonality analysis with hopelessness, rumination/brooding, and dysfunctional attitudes used as predictors and depressive symptoms as outcome. U1, 2, 3: variance explained uniquely (i.e., specificity) by hopelessness (U1), rumination/brooding (U2), and dysfunctional attitudes (U3), respectively. C1, 2, 3: common variance explained (i.e., overlap) by hopelessness or rumination/brooding (C1), hopelessness or dysfunctional attitudes (C2), and rumination/brooding or dysfunctional attitudes (C3). C4: variance explained by hopelessness or rumination/brooding or dysfunctional attitudes.

Importantly, each partition can be labeled as unique (i.e., *specificity*) and common partitions (i.e., *overlap*; see Fig 1). Unique partitions (U) represent the contributions of each single predictor above and beyond the other predictors in explaining the outcome variables (i.e., specificity), and they mathematically equate to incremental R2 and squared semi-partial correlations. Therefore, the unique partition values can range between 0% (null effect) to 100% (perfect fit). For instance, in Fig 1, U1 indicates the amount of variance that only hopelessness can account for, whereas neither brooding nor dysfunctional attitudes can explain that same part of the variance in the outcome. Common partitions (C) quantify the variance in common accounted for by two or more predictors (i.e., overlap). In detail, C1, C2, and C3 represent the first-order common partitions where only two out of three variables are considered at the same time, while C4 indicates a second-order commonality, with all three variables taken into account simultaneously. For instance, C1 quantifies the amount of variance explained by either hopelessness or brooding, but not by dysfunctional attitudes, while C4 indicates the exact amount of variance that is explained by all three of the variables.

Importantly, unlike unique partitions, the common partition may assume both positive and negative values, with the latter suggesting the presence of suppressor predictor variables [25]. Moreover, it is possible to sum all the common partitions involving a specific predictor in order to signify the variance that the predictor shares with the other predictors (i.e., *general overlap*). Thus, by decomposing the predictors’ influence in detailed fashion, CA outperforms other techniques addressing multicollinearity [25], by precisely localizing the site and magnitude of overlap (i.e., multicollinearity).

In line with current guidelines calling for bootstrapping [33, 36, 37], in our study we adopted percentile-based 95% two-tailed bootstrap confidence intervals (1000 bootstrap samples), whenever possible (see below). More specifically, bootstrap estimation was used for a two-fold purpose: i) quantifying the precision of each partition rather than for significance testing [38]; and ii) testing the statistical difference of each partition from the other partitions. In this latter case, for every bootstrap sample, we determined the difference between any two partitions and, across the 1000 bootstrap samples, we obtained the empirical distribution of such difference. Then, we estimated the 95% confidence interval (i.e., CI), with the null hypothesis being rejected if bootstrap CI does not contain zero. All the analyses are conducted in R 3.2.2, using the *yhat* 2.0 package [36].

In accordance with recent guidelines encouraging secondary analyses of published data [39, 40], we evaluated the robustness of our main findings in clinical samples in two published studies in which similar measures were used and the correlation matrices were reported. On the one hand, the availability of the raw data is not necessary to apply CA, given that the correlation matrix serves as foundation for all analytic methods within the general linear model [40]. On the other hand, though, without any information about the single observation level, it is not advisable to estimate bootstrap CIs, which were therefore omitted in our study.

## Results

Pearson’s correlations and multiple regressions with hopelessness, rumination/brooding, and dysfunctional attitudes accounting for depressive (or anxiety) symptoms are reported in S1 and S2 Tables.

### Commonality analysis

**Fig 2:**
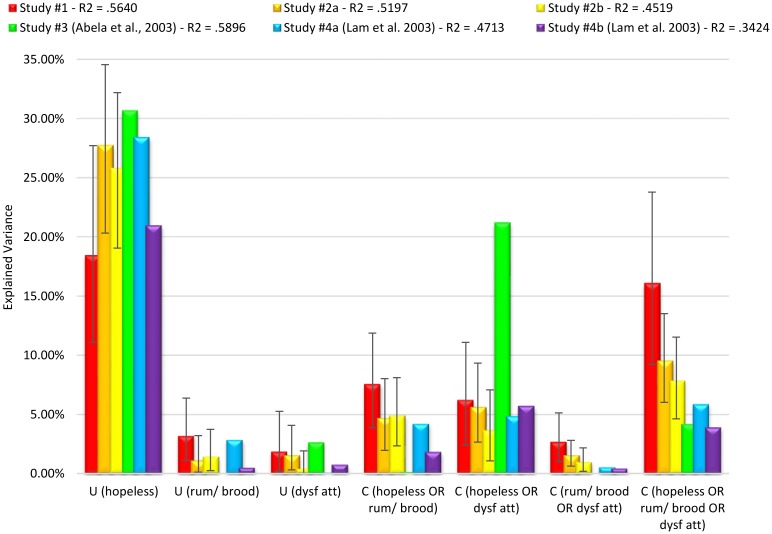
Commonality analysis across Study #1, #2a, b, #3, and #4a, b. Percentile-based 95% bootstrap confidence intervals are reported.

In Study #1, 56.40% of variance related to depressive symptoms (BDI-II) was explained by hopelessness, rumination/brooding, and dysfunctional attitudes, although to a different degree, in that the three cognitive risk factors accounted overall for 48.51%, 29.74%, and 27.04%, respectively (S1 Table, upper part). However, CA revealed a more fine-grained underlying structure (see Fig 2 and Table 1). Interestingly, hopelessness emerged as the most specific factor since its unique contribution was the greatest component of the model (18.46% [CIs: 11.08%; 27.72%]). On the contrary, the unique contributions of rumination/brooding and dysfunctional attitudes were negligible, namely 3.25% [1.12%; 6.37%] and 1.90% [0.17%; 5.25%], respectively. The second largest component was the second-order commonality (C4 in Fig 1), namely the amount of variance explained by hopelessness, rumination/brooding, or dysfunctional attitudes (16.11% [9.23%; 23.79%]). Importantly, the unique component of hopelessness (U1) and the second-order commonality (C4) were statistically significantly different from all the other partitions, but not from each other ([-10.71%; 16.52%], see Table 1). In sum, the CA revealed an overall pattern of overlap between the risk factors, with a clear disproportion between unique (i.e., *specificity*) and common variance (i.e., *general overlap*) at single predictor level in favor of the latter (e.g., rumination/brooding: 3.25 [1.12%; 6.37%] % vs. 26.49% [17.68%; 35.32%], dysfunctional attitudes: 1.90% [0.17%; 5.25%] vs. 25.14% [14.44%; 36.30%]), with the noticeable exception of hopelessness (18.46% [11.08%; 27.72%] vs. 30.05% [20.33%; 39.94%]).

**Table 1:** Commonality analysis of Study #1 and bootstrap-based comparisons among the partitions.

Depressive symptoms (BDI-II)	Partitions	Variance %	95% Boot CIs	#—(2) [95% Boot CIs]	#—(3) [95% Boot CIs]	#—(4) [95% Boot CIs]	#—(5) [95% Boot CIs]	#—(6) [95% Boot CIs]	#—(7) [95% Boot CIs]
(1)	U1 (*hopeless*)	18.46	11.08, 27.72	**6.36, 25.61**	**7.26, 26.70**	**2.43, 21.50**	**3.28, 22.60**	**7.08, 25.98**	-10.71, 16.52
(2)	U2 (*rum/brood*)	3.25	1.12, 6.37	-	-3.01, 5.47	**-8.25, -0.59**	-9.18, 2.61	-2.01, 3.84	**-21.07, -5.43**
(3)	U3 (*dysf att*)	1.90	0.17, 5.25		-	**-10.59, -0.14**	**-8.44, -0.87**	-2.56, 1.93	**-21.46, -7.65**
(4)	C1 (*hopeless or rum/brood*)	7.64	3.88, 11.86			-	-6.31, 8.30	**0.01, 9.79**	**-16.99, -0.26**
(5)	C2 (*hopeless or dysf att*)	6.30	2.40, 11.09				-	-0.47, 8.41	**-17.05, -3.54**
(6)	C3 (*rum/brood or dysf att*)	2.74	1.03, 5.12					-	**-20.69, -7.13**
(7)	C4 (*hopeless or rum/brood or dysf att*)	16.11	9.23, 23.79						-

*Note*. Percentile-based 95% bootstrap confidence intervals. Bold bootstrap confidence intervals indicate a statistically significant difference at α = 0.05. U1, U2, and U3: unique partitions; C1, C2, C3, C4: common partitions.

### Replication, convergent validity, and depression-specificity

Given the exploratory nature of our study, we tried to confirm the robustness of its main findings in an independent sample and across different measures. We did so in Study #2, where we aimed to confirm Study #1 (S1 Table, middle part). Fig 2 and Table 2 (upper part) show the unique component of hopelessness was the greatest partition (U1; 27.76% [20.32%; 34.56%]), followed by the second-order commonality (C4; 9.58% [6.01%; 13.52%]), in explaining depressive symptoms (BDI-II; 51.97%). Moreover, the amount of variance uniquely explained by hopelessness was statistically greater than all the other partitions, and this speaks in favor of considering the unique component of hopelessness as an important risk factor for depression. Next to this finding, a general pattern of overlap clearly emerged for rumination/brooding (unique 1.13% [0.11%; 3.20%] vs. general overlap 15.87% [10.49%; 21.80%]) and dysfunctional attitudes (unique 1.57% [0.30%; 4.08%] vs. general overlap 16.80% [11.16%; 23.06%]), but not for hopelessness (unique 27.76% [20.32%; 34.56%] vs. general overlap 19.95% [13.74%; 26.06%]).

**Table 2:** Commonality analysis of Study #2a and #2b and bootstrap-based comparisons among the partitions.

BDI-II	Partitions	Variance %	95% Boot LL and UL CCs	#—(2) [95% Boot LL and UL CIs]	#—(3) [95% Boot LL and UL CIs]	#—(4) [95% Boot LL and UL CIs]	#—(5) [95% Boot LL and UL CIs]	#—(6) [95% Boot LL and UL CIs]	#—(7) [95% Boot LL and UL CIs]
(1)	U1 (*hopeless*)	27.76	20.32, 34.56	**18.34, 34.01**	**17.61, 33.27**	**14.79, 30.87**	**13.56, 29.72**	**18.35, 33.63**	**9.76, 26.08**
(2)	U2 (*rum/brood*)	1.13	0.11, 3.20	-	-3.51, 2.37	**-6.50, -1.09**	**-8.72, -0.51**	-1.61, 1.44	**-12.61, -4.59**
(3)	U3 (*dysf att*)	1.57	0.30, 4.08		-	-6.92, 1.19	-7.34, 0.87	-1.22, 2.32	**-11.93, -3.91**
(4)	C1 (*hopeless or rum/brood*)	4.72	1.96, 8.03			-	-6.57, 4.53	**0.16, 6.63**	-9.07, 0.90
(5)	C2 (*hopeless or dysf att*)	5.65	2.65, 9.34				-	**0.77, 7.89**	-8.16, 0.22
(6)	C3 (*rum/brood or dysf att*)	1.57	0.62, 2.80					-	**-11.78, -4.43**
(7)	C4 (*hopeless or rum/brood or dysf att*)	9.58	6.01, 13.52						-
DASS-Dep	Partitions	Variance %	95% Boot LL and UL CCs	#—(2) [95% Boot LL and UL CIs]	#—(3) [95% Boot LL and UL CIs]	#—(4) [95% Boot LL and UL CIs]	#—(5) [95% Boot LL and UL CIs]	#—(6) [95% Boot LL and UL CIs]	#—(7) [95% Boot LL and UL CIs]
(1)	U1 (*hopeless*)	25.84	19.05, 32.20	**16.40, 31.28**	**18.26, 31.95**	**13.85, 28.13**	**14.91, 28.93**	**17.40, 31.61**	**10.34, 25.13**
(2)	U2 (*rum/brood*)	1.48	0.24, 3.73	-	-1.23, 3.44	**-6.35, -0.65**	-6.09, 1.72	-0.80, 2.60	**-10.22, -2.71**
(3)	U3 (*dysf att*)	0.43	0.00, 1.91		-	**-7.94, -1.12**	**-5.93, -0.87**	-1.37, 0.67	**-10.94, -4.13**
(4)	C1 (*hopeless or rum/brood*)	4.92	2.33, 8.11			-	-4.08, 6.24	**0.94, 7.46**	-6.85, 1.08
(5)	C2 (*hopeless or dysf att*)	3.66	1.07, 7.08				-	**0.37, 5.87**	**-7.56, -0.92**
(6)	C3 (*rum/brood or dysf att*)	0.99	0.19, 2.16					-	**-10.31, -3.74**
(7)	C4 (*hopeless or rum/brood or dysf att*)	7.86	4.63, 11.54						-

*Note*. Percentile-based 95% bootstrap confidence intervals. Bold bootstrap confidence intervals indicate a statistically significant difference at α = 0.05. U1, U2, and U3: unique partitions; C1, C2, C3, C4: common partition

We then strengthened the validity of our results by replacing our BDI-II outcome measure with another validated measure of depressive symptoms (DASS-Dep; Study #2b, R^2^ = .4519; S1 Table, lower part). Supporting the robustness of our approach, very similar results were obtained (see Fig 2). The overall pattern of overlap for the risk factors clearly emerged (Table 2, lower part) along with the primary role played by the unique component of hopelessness (25.84% [19.05%; 32.20%]).

Finally, considering the comorbidity of depression, anxiety, and stress-related symptoms [41], we investigated whether the unique component of hopelessness is a specific marker of depression, compared to other distress symptoms (S2 Table, upper part). It is noteworthy that, even after controlling for anxiety and stress symptom levels, the unique component of hopelessness still played a major role (14.31% [9.79%; 19.25%]) compared to the other factors’ unique components, ranging from 0% [0%; 0.052%] for dysfunctional attitudes to 5.43% [3.04%; 8.71%] for stress symptom levels. All the predictors explained a significant amount of variance via commonalities, ranging from 12.95% for dysfunctional attitudes to 32.21% for stress symptoms. Supporting the specificity of the unique component of hopelessness, its contribution was null in explaining either anxiety (0.10% [0%; 0.96%]) or stress levels (0.06% [0%; 0.67%]), after controlling for depression (middle and lower part of S2 Table).

### Clinical extension in depressed individuals

Previous research shows many aspects of depression lie on a continuum between healthy/subclinical and clinically depressed individuals (continuity hypothesis; [42]), while other features may differ between the two groups in a categorical manner ([43]). Therefore, in order to explore whether the structure of cognitive vulnerability unveiled in healthy/subclinical individuals extends to depressed patients too, we performed a secondary analysis on two additional, published studies (Study #3: [28]; Study #4a,b: [21]). In both studies similar measures of the cognitive risk factors were administered in clinically depressed samples along with self-report (Study #3 and #4a) and clinical interview-based (Study #4b) measures of depressive symptoms (Table 3). The CA confirmed that, in explaining depressive symptoms (Study #3: 58.96%; Study #4a: 47.16%; Study #4b: 34.24%), hopelessness plays a major role (Study #3: unique 30.69% vs. general overlap 25.56%; Study #4a: unique 28.42% vs. general overlap 15.14%; %; Study #4b: unique 20.97% vs. general overlap 11.52%), compared to rumination (Study #3: unique 0.02% vs. general overlap 4.39%; Study #4a: unique 2.91% vs. general overlap 10.78%; Study #4b: unique 0.52% vs. general overlap 6.24%) and dysfunctional attitudes (Study #3: unique 2.65% vs. general overlap 25.44%; Study #4a: unique 0.12% vs. general overlap 11.44%; Study #4b: unique 0.78 vs. general overlap 10.11%). For these latter variables, the analysis confirmed their lack of specificity (see Fig 2). Moreover, the inspection of each single partition did not reveal any prominent locus of major overlap (i.e., multicollinearity), with the exception of the overlap between hopelessness and dysfunctional attitudes in Study #3. Although interesting, this specific finding should be interpreted with caution, as it was deviant from the other included studies and emerged in a sample of composite nature (i.e., individuals with no diagnosis, MDD, and MDD with borderline personality).

**Table 3:** Commonality analysis on published studies Study #3, #4a, and #4b.

Partitions	Study #3 (Abela et al., 2003; BDI; R^2^ = 0.5896)	Study #4a (Abela et al., 2003; BDI; R^2^ = 0.4716)	Study #4b (Abela et al., 2003; HAM-D; R^2^ = 0.3424)
	Variance %	Variance %	Variance %
U1 (*hopeless*)	30.69	28.42	20.97
U2 (*rum/brood*)	0.02	2.91	0.52
U3 (*dysf att*)	2.65	0.12	0.78
C1 (*hopeless or rum/brood*)	0.16	4.27	1.86
C2 (*hopeless or dysf att*)	21.21	4.93	5.73
C3 (*rum//brood or dysf att*)	0.03	0.57	0.46
C4 (*hopeless or rum/brood or dysf att*)	4.20	5.94	3.92

*Note*. U1, U2, and U3: unique partitions; C1, C2, C3, C4: common partitions.

### Symptoms-level commonality analysis

**Fig 3:**
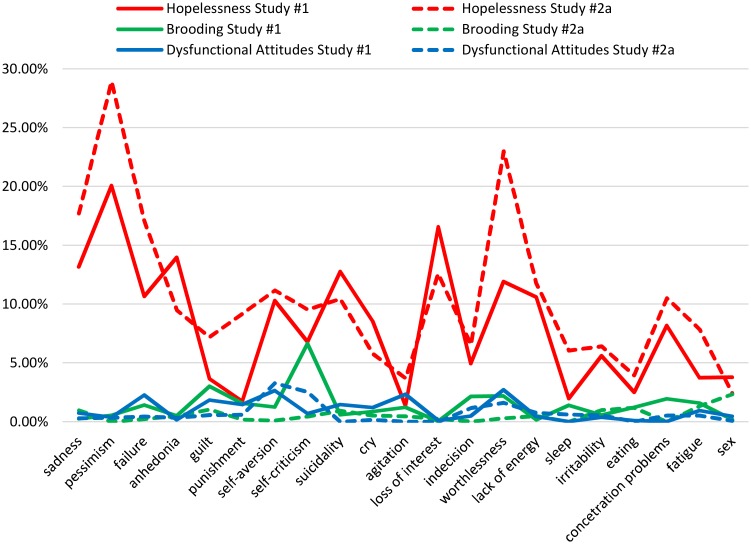
Variance explained uniquely (specificity) by hopelessness (U1), rumination/brooding (U2), and dysfunctional attitudes (U3) in Study #1 and #2a.

Recently, research has stressed the heterogeneity of the depressive syndrome [44–47], with different psychosocial risk factors being associated to different depressive symptoms [48]. In line with this new perspective, we explored whether the unique component (i.e., *specificity*) and their common variance (i.e., *general overlap*) of cognitive risk factors are differently related to different depressive symptoms. Therefore, we performed CA on both Study #1 and #2a at the level of the 21 depressive symptoms, as measured by the BDI-II [48]. The analysis confirmed a differential pattern for the unique partition of hopelessness compared to the ones of rumination and dysfunctional attitudes (Fig 3). Interestingly, in both studies, the symptom that the unique component of hopelessness predicted the most was *pessimism* (20.08% and 28.94%). Moreover, by setting a cut-off of at least 9% explained variance (moderate effect; [35]), hopelessness significantly explained the variance associated with a subset of depressive symptoms in Study #1 and #2a, namely *sadness* (13.16% and 17.71%), *sense of failure* (10.67% and 17.08%), *anhedonia* (13.98% and 9.47%), *self-aversion* (10.29% and 11.17%), *suicidality* (12.77% and 10.45%), *loss of interest* (16.57% and 12.60%), *worthlessness* (11.91% and 23.01%), *lack of energy* (10.59% and 11.76%), and *concentration problems* (10.52% only in study #2a).

**Fig 4:**
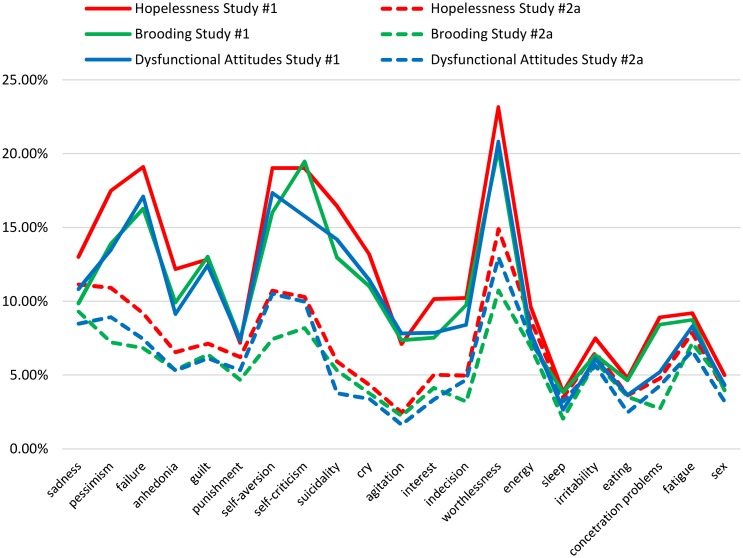
General overlap of hopelessness, rumination/brooding, and dysfunctional attitudes in accounting for the single depressive symptoms in Study #1 and #2a.

With respect to the general overlap present in each cognitive risk factor, there was a consistent pattern across risk factors at the level of each depressive symptom in both Study #1 and #2a (Fig 4). Interestingly, across the three cognitive risk factors in both studies, only the *worthlessness* symptom was accounted for at least 9%, thus suggesting that this symptom is common to all three cognitive risk factors, rather than specific to only one of them.

Finally, we explored whether the important effect of specificity for the unique component of hopelessness was only due to the similarity in content with the pessimism-related item part of the outcome measure (i.e., BDI-II). To rule out this potential confound, we re-ran the CA on the depressive symptoms scale (i.e., BDI-II total score) in both Study #1 and #2a, after excluding the pessimism-related item. The analysis revealed very similar results for hopelessness compared to the prior analysis (unique 17.40% [10.18%; 26.68%] vs. general overlap 29.43% [19.65%; 39.09%] in Study #1; unique 25.82% [18.34%; 32.57%] vs. general overlap 19.37% [13.28%; 25.36%] in Study #2a).

## Discussion

Cognitive vulnerability to depression is a complex domain in mental health research, where there is evidence for multiple, interrelated factors that are well-validated predictors of depression. Nevertheless, it is still unclear to what extent different risk factors overlap or, on the contrary, show specificity in accounting for depression. To address this issue, we compared three well-established cognitive risk factors (i.e., dysfunctional attitudes, hopelessness, and rumination/brooding) by adopting an innovative analytical approach (i.e., commonality analysis, CA; [27]) that allowed us to estimate the degree of specificity and overlap of key cognitive vulnerability factors in explaining depressive symptoms.

In a series of original studies and secondary analyses on published studies, CA revealed a dominant role of hopelessness in uniquely accounting for depressive symptoms. This finding held in both healthy and clinically depressed samples, with the unique component of hopelessness emerging as depression-specific rather than common to anxiety and stress too. Interestingly, when probed at the level of single depressive symptoms, the unique component of hopelessness first and foremost predicted pessimism, along with other symptoms, such as sadness, suicidality, self-aversion, worthlessness, loss of interest, lack of energy, and anhedonia. It is noteworthy that these are many of the symptoms hypothesized to be part of the hopelessness depression syndrome by the Hopelessness Theory [9]. Altogether, these findings suggest that a detrimental and gloomy view of the future represents a powerful factor facilitating and, perhaps, maintaining depression. These results can be linked to a recent theoretical perspective that frames future-thinking as a cause rather than as a correlate or symptom of depression [11]. More specifically, Roepke and Seligman proposed that with respect to depression “[…] faulty prospection is the primary cause: That it accounts for more of the variance in MDD than other causes such as the negative view of the self, negative view of the world, and pessimistic explanatory style” [11]. Although no such strong causal claims can be derived from the present studies given their cross-sectional nature, our results suggest that distorted future-thinking plays a pivotal role in depression, a fact that has important consequences for research and clinical practice (see below).

Surprisingly, the CA did not reveal any substantial unique contribution of either rumination/brooding or dysfunctional attitudes in explaining depressive symptoms. These two factors did account for depression, but mostly via non-specific contributions (i.e., common partitions), thus implying an unexpected level of overlap of rumination and dysfunctional attitudes when compared to hopelessness in explaining depression. Although striking, this finding does not imply that rumination and dysfunctional attitudes are constructs to abandon, but rather that (some of) their features are likely to be transversal to other cognitive risk factors. For instance, whereas a central feature of rumination is usually taken to be its repetitiveness [49], this may potentially be present in hopelessness and dysfunctional attitudes too, thus suggesting the existence of hybrid types of thoughts (i.e., repetitive hopeless thoughts; [50]).

As already mentioned, the three risk factors analyzed in our studies showed a substantial level of general overlap among one another. When we tried to qualify this general overlap component by running CA for single depressive symptoms, worthlessness emerged as the symptom that was explained the most by the three risk factors in a non-specific way (i.e., general overlap). This suggests that part of the predictive power that each of them share with others is likely to be their mapping onto a negative view of the self. Worthlessness is indeed considered to be one of the main hubs of depression [4, 5, 7]; therefore, it is reasonable to assume that worthlessness is a primary depressive feature that is captured by a variety of cognitive risk factors.

Altogether, our studies suggest that a negative view of the future (i.e., hopelessness) and the self (i.e., worthlessness) potentially represent major risks in facilitating and, perhaps, maintaining depression. Interestingly, important theoretical models have already underlined the pivotal role of the self-future axis in depression. In their survey-based study on the social origins of depression, Brown and Harris [51] found evidence that, when exposed to stress, low self-esteem impedes coping adaptively with stress and, in turn, leads to generalized feelings of hopelessness, a key marker for depression. In line with this, the Hopelessness Theory [9] posits that hopelessness is rooted in specific attributional styles, such as the one whereby the self is viewed as intrinsically flawed and disvalued in reaction to negative events. More recently, Roepke and Seligman [11] proposed that, in the context of depression, the self and the future are very tightly interconnected and hardly (though not impossibly) distinguishable, in that “[i]f an individual believed that he/she was unlovable, but only for today, this would not be so discouraging; the thought ‘I am unlovable’ is distressing because it implies ‘no one will *ever* love me’ (p. 15). In sum, our results highlight the crucial importance of the view of the future and the self in accounting for depression, a fact that leads to important clinical implications.

Although standard cognitive-behavioral therapy entails a few techniques that directly or indirectly address negative future-thinking biases (for a review, see [11]), our results suggest that this variable should be routinely assessed and targeted in interventions. With respect to the latter, several new treatment packages have been developed to target negative future-thinking, such as the future-directed therapy [52], goal-setting and planning [53], and hope therapy [54]. More basic and clinical research is needed, but it is noteworthy that the latter intervention has been reported to improve both hope and self-esteem, beyond improving individual levels of depression and anxiety.

Our study is characterized by several limitations that we address here. First, almost all of the measurements were obtained with self-report questionnaires. This raises the concern about whether a common-method bias might have affected the relationship among the variables (i.e., inflated or deflated correlations; [55]). Although legitimate, this concern is notably mitigated by the fact that similar results were obtained when depressive symptoms were measured with a validated clinician-based interview (Study #4b), thus strengthening the validity of our results. Moreover, it is important to remember that for most of the constructs considered in our studies, no information-processing paradigm has been developed yet [56], while well-validated self-report questionnaires are readily available. Second, since we used convenience samples in both Study #1 and #2, it is possible that the observations in our samples were not independent (i.e., undergraduate students). On the one hand, violation of the independence assumption does not alter the estimated regression coefficients and model fit, but rather impacts their statistical inference (i.e., standard errors and *p*-values). As the CA primarily relies on the model fit, the presented partitions are still valid. On the other hand, the bootstrap-based confidence intervals assume independence among observations and may not capture the true variability when this assumption is violated. Therefore, although we expect negligible correlation in the samples, the reported CIs may not have the appropriate coverage. Third, no alpha adjustment for multiple comparisons was performed. It is worth stressing that, although this may have inflated the rate of false positives at level of bootstrap confidences intervals, the strong robustness of our results across multiple studies suggests that the reported findings are to be considered trustworthy. Fourth, all our studies were correlational; therefore, the presented findings are to be taken as suggesting associations, rather than implying causality. This being said, we encourage replicating our results from a temporal perspective; this being supported by the fact that hopelessness/negative future-thinking can predict future depressive symptoms [57–59].

In closing, we believe that our approach is promising and could fruitfully be applied to many research questions. Needless to say, researchers could address a similar question as the one we proposed in other disorders, such as anxiety disorders. It is also possible to extend our analysis by including risk factors of different orders, such as neurophysiological parameters, genotypes, and social variables. By doing that, we could better integrate our investigations by potentiating a biopsychosocial perspective. Finally, a promising application of CA in mental health research could be its integration with meta-analytical investigations, given the low threshold of required information for its application (i.e., correlation matrix; [39]).

## Supporting Information

S1 Dataset Study #1. (CSV)

S2 Dataset Study #2. (CSV)

S1 Table Pearson’s correlations, regression coefficients, and commonality analysis of Study #1, #2a, and #2b with levels of hopelessness, rumination (brooding), and dysfunctional attitudes explaining depressive symptoms. (PDF)

S2 Table Pearson’s correlations, regression coefficients, and commonality analysis of Study #2b with levels of hopelessness, rumination (brooding), dysfunctional attitudes, explaining depressive symptoms. (PDF)
