# Supplementary material for: Unveiling the Structure of Cognitive Vulnerability for Depression: Specificity and Overlap
Source: PLoS One. 2016 Dec 16;11(12):e0168612. doi: 10.1371/journal.pone.0168612 (PMC5161451; doi:10.1371/journal.pone.0168612)
Supplement: S1 Table — (PDF) [file pone.0168612.s003.pdf]

S1 Table. Pearson's correlations, regression coefficients, and commonality analysis of Study #1, #2a, and #2b with levels of hopelessness, rumination (brooding), and dysfunctional attitudes explaining depressive symptoms

Depressive Symptoms (BDI-II; Study #1, R^2 = .5640)	Predictor	r_1	r_2	r_y	*B*	*SE B*	β	*t*	*p*	Unique variance / *Specificity*	Common variance / *General overlap*	Total variance
	Hopelessness (1)	-		.697	1.139	.101	.513	11.269	< .001	18.46%	30.05%	48.51%
	Rumination (2)	.474	-	.545	.511	.108	.219	4.731	< .001	3.25%	26.49%	29.74%
	Dysfunctional Attitudes (3)	.475	.498	.520	.045	.012	.167	3.612	< .001	1.90%	25.14%	27.04%
Depressive Symptoms (BDI-II; Study #2a, R^2 = .5197)	Predictor	r_1	r_2	r_y	*B*	*SE B*	β	*t*	*p*	Unique variance / *Specificity*	Common variance / *General overlap*	Total variance
	Hopelessness (1)	-		.691	1.214	.072	.588	16.778	< .001	27.76%	19.95%	47.71%
	Rumination (2)	.377	-	.412	.285	.084	.123	3.381	< .001	1.13%	15.87%	17.00%
	Dysfunctional Attitudes (3)	.384	.462	.429	.040	.010	.146	3.985	< .001	1.57%	16.80%	18.37%
Depressive Symptoms (DASS-Dep; Study #2b, R^2 = .4519)	Predictor	r_1	r_2	r_y	*B*	*SE B*	β	*t*	*p*	Unique variance / *Specificity*	Common variance / *General overlap*	Total variance
	Hopelessness (1)	-		.650	.565	.037	.568	15.154	< .001	25.84%	16.45%	42.29%
	Rumination (2)	.377	-	.391	.158	.043	.141	3.624	< .001	1.48%	13.77%	15.25%
	Dysfunctional Attitudes (3)	.384	.462	.360	.010	.005	.077	1.965	< .050	0.43%	12.52%	12.95%

Note. r_y: Pearson's correlation between a predictor and the outcome (depressive symptoms). Study #1: *n* = 304 and Study #2a,b: *n* = 491. Total variance represents the amount of variance explained by each single predictor in a univariate fashion and it equates to the sum of unique variance (*specificity*) and common variance (*general overlap*).
