# Supplementary material for: Unveiling the Structure of Cognitive Vulnerability for Depression: Specificity and Overlap
Source: PLoS One. 2016 Dec 16;11(12):e0168612. doi: 10.1371/journal.pone.0168612 (PMC5161451; doi:10.1371/journal.pone.0168612)
Supplement: S2 Table — (PDF) [file pone.0168612.s004.pdf]

S2 Table. Pearson's correlations, regression coefficients, and commonality analysis of Study #2b with levels of hopelessness, rumination (brooding), dysfunctional attitudes, explaining depressive symptoms

Depressive symptoms (DASS-Dep, R^2 = .5732)	Predictor	r_1	r_2	r_3	r_4	r_y	*B*	*SE B*	β	*t*	*p*	Unique variance / *Specificity*	Common variance / *General overlap*	Total variance
	Hopelessness (1)	-				.650	.442	.035	.444	12.750	< .001	14.31%	27.98%	42.29%
	Rumination (2)	.377	-			.391	.059	.040	.053	1.501	.134	0.20%	15.05%	15.25%
	Dysfunctional Attitudes (3)	.384	.462	-		.360	.000	.005	.001	0.024	.981	0%	12.95%	12.95%
	Anxiety (4)	.380	.286	.328	-	.514	.137	.041	.129	3.345	< .001	0.98%	25.46%	26.44%
	Stress (5)	.426	.414	.379	.625	.614	.263	.033	.321	7.855	< .000	5.43%	32.21%	37.64%
**Anxiety-related symptoms (DASS-Anx, R^2 = .4245)**	**Predictor**	r_1	r_2	r_3	r_4	r_y	*B*	*SE B*	β	*t*	*p*	Unique variance / *Specificity*	Common variance / *General overlap*	Total variance
	Hopelessness (1)	-				.380	.040	.044	.043	.919	.358	0.10%	14.36%	14.46%
	Rumination (2)	.377	-			.286	-.038	.043	-.036	-.884	.377	0.09%	8.08%	8.17%
	Dysfunctional Attitudes (3)	.384	.462	-		.328	.010	.005	.082	.2025	< .050	0.49%	10.27%	10.76%
	Depression (4)	.650	.391	.360	-	.514	.164	.049	.174	3.345	< .001	1.33%	25.11%	26.44%
	Stress (5)	.426	.414	.379	.614	.625	.373	.035	.483	10.650	< .001	13.46%	25.57%	39.03%
**Stress-related symptoms (DASS-Stress, R^2 = .5329)**	**Predictor**	r_1	r_2	r_3	r_4	r_y	*B*	*SE B*	β	*t*	*p*	Unique variance / *Specificity*	Common variance / *General overlap*	Total variance
	Hopelessness (1)	-				.426	-.039	.051	-.032	-.768	.443	0.06%	18.13%	18.19%
	Rumination (2)	.377	-			.414	.196	.050	.144	3.944	< .001	1.50%	15.61%	17.11%
	Dysfunctional Attitudes (3)	.384	.462	-		.379	.011	.006	.070	1.901	.058	0.35%	14.02%	14.37%
	Depression (4)	.650	.391	.360	-	.614	.429	.055	.351	7.855	< .001	5.94%	31.70%	37.64%
	Anxiety (5)	.380	.286	.328	.514	.625	.509	.048	.392	10.650	<.001	10.92%	28.11%	39.03%

Note. r_y: Pearson's correlation between a predictor and the outcome *n* = 491. Total variance represents the amount of variance explained by each single predictor in a univariate fashion and it equates to the sum of unique variance (*specificity*) and common variance (*general overlap*).
